# Nuclear LEF1/TCF4 correlate with poor prognosis but not with nuclear β-catenin in cerebral metastasis of lung adenocarcinomas

**DOI:** 10.1007/s10585-012-9552-7

**Published:** 2012-12-08

**Authors:** A. Bleckmann, L. Siam, F. Klemm, E. Rietkötter, Chr. Wegner, F. Kramer, T. Beissbarth, C. Binder, Chr. Stadelmann, T. Pukrop

**Affiliations:** 1 Department of Hematology/Oncology, University Medical Center Göttingen, 37099 Göttingen, Germany; 2 Department of Neurosurgery, University Medical Center Göttingen, 37099 Göttingen, Germany; 3 Department of Medical Statistics, University Medical Center Göttingen, 37099 Göttingen, Germany; 4 Department of Neuropathology, University Medical Center Göttingen, 37099 Göttingen, Germany

**Keywords:** Lung cancer, Brain metastases, LEF1, TCF4, Ki67, β-catenin, TTF1

## Abstract

An essential function of the transcription factors LEF1/TCF4 in cerebral metastases of lung adenocarcinomas has been described in mouse models, suggesting a WNT/β-catenin effect as potential mechanism. Their role in humans is still unclear, thus we analyzed LEF1, TCF4, β-catenin, and early stage prognostic markers in 25 adenocarcinoma brain metastases using immunohistochemistry (IHC). IHC revealed nuclear TCF4 in all adenocarcinoma samples, whereas only 36 % depicted nuclear LEF1 and nuclear β-catenin signals. Samples with nuclear LEF1 as well as high TCF4 (++++) expression were associated with a shorter survival (*p* = 0.01, HR = 6.68), while nuclear β-catenin had no significant impact on prognosis and did not significantly correlate with nuclear LEF1. High proliferation index Ki67 was associated with shorter survival in late-stage disease (*p* = 0.03, HR 3.27). Additionally, we generated a LEF1/TCF4 as well as an AXIN2 signature, the latter as representative of WNT/β-catenin activity, following a bioinformatics approach with a gene expression dataset of cerebral metastases in lung adenocarcinoma. To analyze the prognostic relevance in primary lung adenocarcinomas, we applied both signatures to a microarray dataset of 58 primary lung adenocarcinomas. Only the LEF1/TCF4 signature was able to separate clusters with impact on survival (*p* = 0.01, HR = 0.32). These clusters displayed diverging enrichment patterns of the cell cycle pathway. In conclusion, our data show that LEF1/TCF4, but not β-catenin, have prognostic relevance in primary and cerebrally metastasized human lung adenocarcinomas. In contrast to the previous in vivo findings, these results indicate that LEF1/TCF4 act independently of β-catenin in this setting.

**Electronic supplementary material:**

The online version of this article (doi:10.1007/s10585-012-9552-7) contains supplementary material, which is available to authorized users.

## Introduction

Lung cancer is the leading cause of cancer death worldwide due to its aggressive clinical course coupled with a high frequency of distant metastasis and insufficient treatment options [1]. In 10–25 % of lung cancer patients, brain metastases are already present at diagnosis, and a further 40–50 % develop brain metastasis during the course of treatment. Furthermore, primary lung cancers are responsible for 40–50 % of all brain metastases. Patients with lung cancer brain metastases have an unfavorable prognosis, since micro-metastases are shielded by the blood brain barrier and are frequently resistant to radio- and/or chemotherapy. However, studies on lung cancer cerebral metastases are rare due to the lack of preclinical models and scarceness of histological material. Moreover, clinical trials often exclude patients with cerebral metastases, thus the prognosis has not essentially improved over the last decades [2, 3].

The heterogeneity of primary lung cancers and their varying biological behavior still remain a challenge to pathologists and oncologists [4]. Common histological classification defines two main groups: small cell lung cancer (SCLC) and non-small cell lung cancer (NSCLC), the latter including adeno-, squamous cell, and large cell carcinoma. This classification does not reflect the differences in biological and clinical behavior sufficiently. At least adenocarcinomas, the most frequent lung cancer subtype, can be further subdivided according to histological and molecular features [4]. Moreover, some of the histological markers in primary lung adenocarcinomas have prognostic impact. TTF1-negativity as well as a high proliferation index Ki67 are associated with poor overall survival in early-stage disease [5–8].

Recent in vivo experiments in a xenograft brain lung metastasis model postulated a functional role for lymphoid enhancer factor 1/T cell factor 4 (LEF1/TCF4) in a subgroup of adenocarcinomas [9]. Both LEF1 and TCF4 are sequence-specific transcription factors of the high mobility group (HMG) family. They can utilize at least two mechanisms to regulate transcriptional activity. On the one hand, the LEF/TCF protein family has the capacity to bend DNA after binding to the minor grove of the double helix. This architectural alteration is necessary for the subsequent interaction with other transcription factors to regulate gene transcription. On the other hand, the members of the LEF/TCF protein family are the transcriptional effectors of WNT/β-catenin signaling [10].

Activation of the WNT/β-catenin pathway, also known as canonical WNT signaling, leads to stabilization of β-catenin. Subsequently, β-catenin translocates into the nucleus, where it binds as a co-activator to the LEF/TCF proteins and regulates transcription of WNT/β-catenin target genes. Given that β-catenin has no DNA binding motif, the LEF/TCF proteins are necessary for the transcriptional consequences of WNT/β-catenin signaling. Apart from WNT signaling through ligand binding, genetic mutations of β-catenin or the β-catenin degradation complex can also lead to stabilization, cytoplasmic accumulation, nuclear translocation, and binding of β-catenin to LEF/TCF transcription factors [11].

Recent mouse studies also demonstrated an impact of a WNT-lung signature on relapse of patients with primary lung adenocarcinomas [9]. This WNT-lung signature was derived from a gene expression analysis with the typical WNT/β-catenin activator WNT3a. A β-catenin gene signature alone [12] was not predictive in this context [9].

We and others recently demonstrated a potential role for WNT signaling in brain metastasis of breast cancer patients [13–15]. Thus, in the present study, we focused on the role of WNT signaling in brain metastases of lung adenocarcinomas, investigating the expression patterns of LEF1, TCF4, and β-catenin in tissue samples of lung adenocarcinoma brain metastases and their impact on patient survival. Furthermore, following a bioinformatics approach we generated a LEF1/TCF4 as well as an AXIN2 gene signature in cerebral metastases and investigated their prognostic value in a dataset of primary lung adenocarcinomas. Moreover, we investigated the prognostic relevance of TTF1 and Ki67 in brain metastasis samples.

## Materials and methods

### Patient samples

Forty brain metastasis samples of lung cancers were collected with informed consent during medically indicated neurosurgical procedures following approval by the local ethics committee. Twenty-five were defined as lung adenocarcinomas and included into further investigations. This patient cohort was characterized in terms of demographics, clinical baseline data, and applied treatment concepts.

### Immunohistochemistry (IHC)

**Fig. 1:**
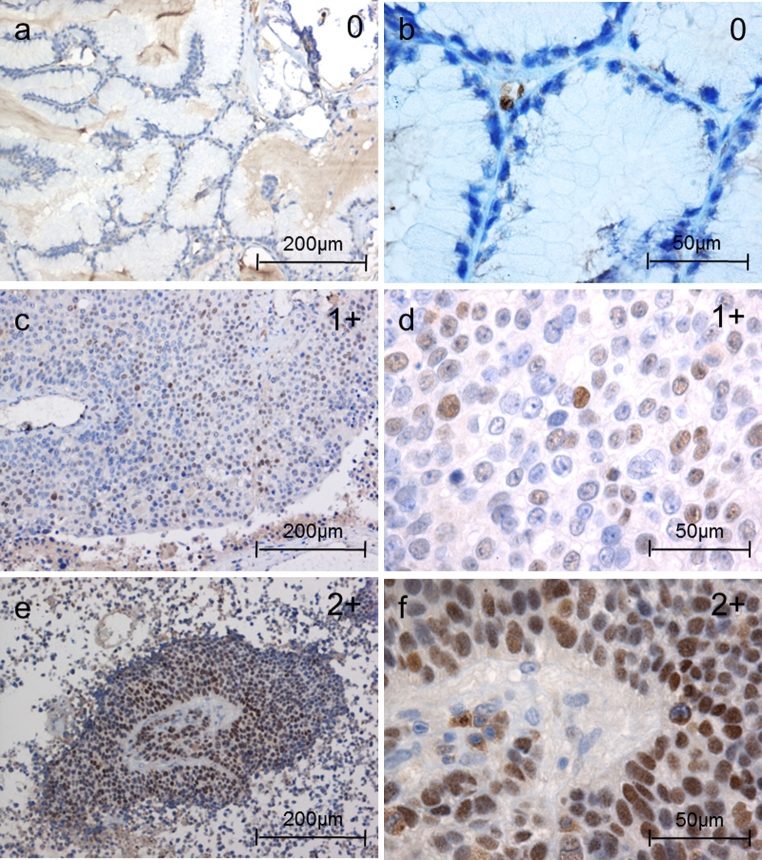
Nuclear LEF1 expression in brain metastases of adenocarcinomas of the lung (IHC): examples of the different LEF1 nuclear patterns. Nuclear LEF1 expression were classified into four groups: 0 = 0 %, 1+ = 1–25 %, 2+ = 25–50 % and 3+ >50 % of the tumor cells with positive nuclear staining

**Fig. 2:**
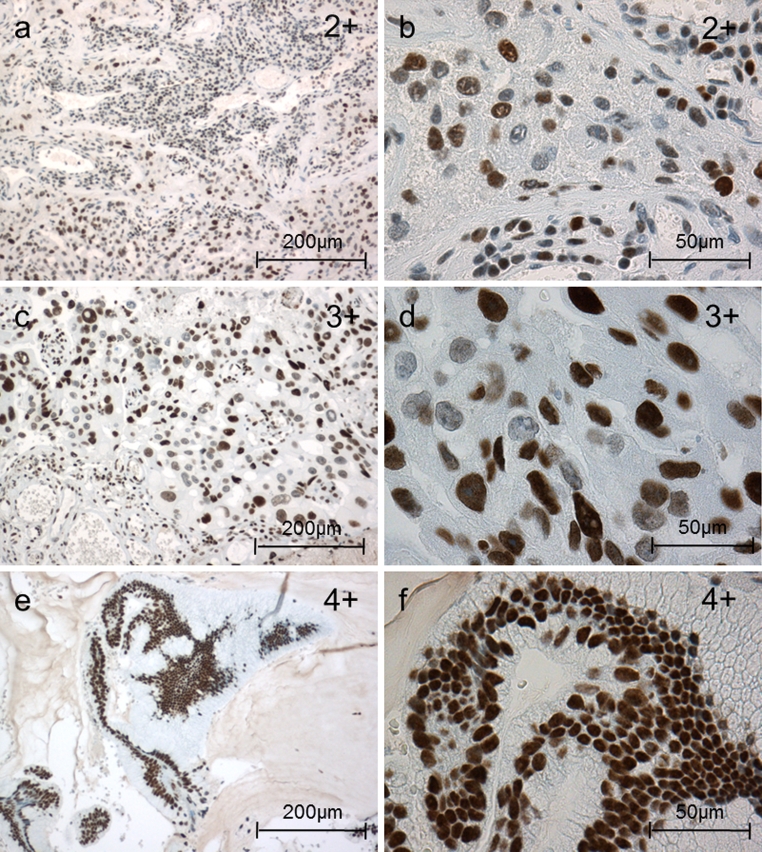
Nuclear TCF4 expression in brain metastases of adenocarcinomas of the lung (IHC): examples of the different TCF4 nuclear patterns. Nuclear TCF4 expression was classified into five groups: 0 = 0 %, 1+ = 1–33 %, 2+ = 33–66 %, 3+ = 66–99 % and 4+ > 99 % of the cells with positive nuclear TCF4 staining

**Fig. 3:**
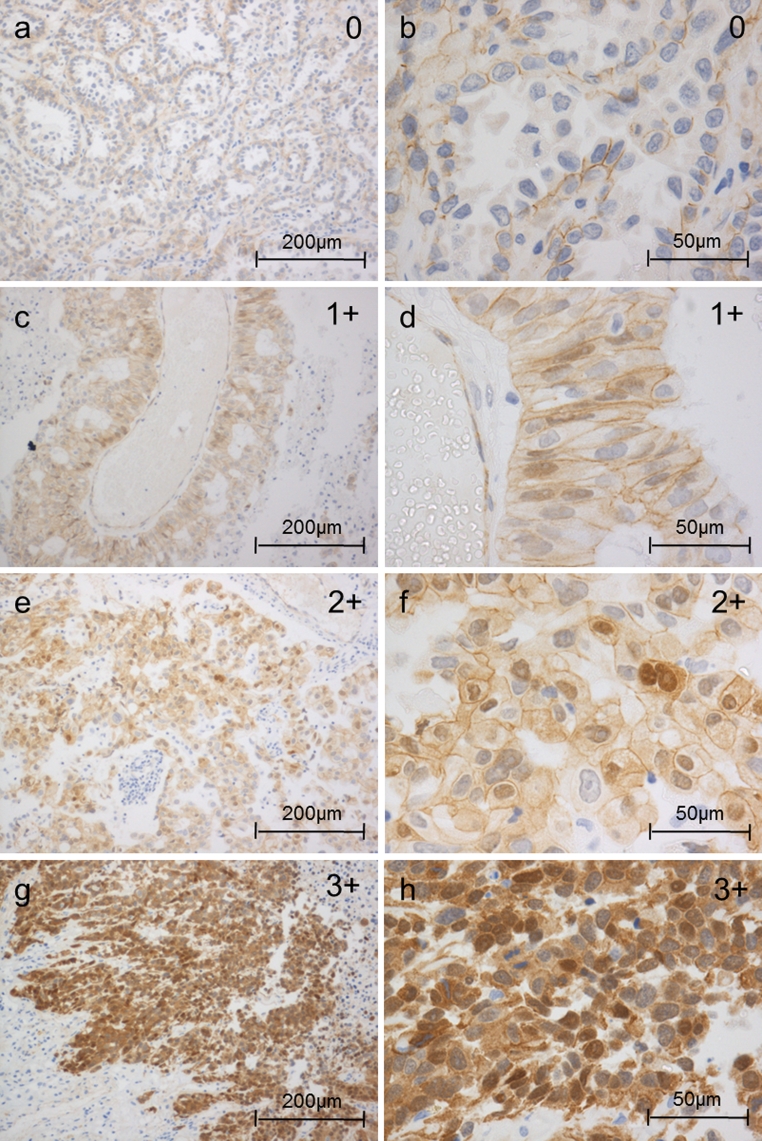
Nuclear β-catenin expression in brain metastases of adenocarcinomas of the lung (IHC): examples of the different nuclear β-catenin patterns. Nuclear β-catenin expression was classified into four groups: 0 = 0 %, 1+ = 1–25 %, 2+ = 25–50 % and 3+ > 50 % of the tumor cells with positive nuclear staining. Cytoplasmic and membrane staining did not enter the grading scheme

For diagnostic purposes, all tissue samples were stained with antibodies to cytokeratin (CK) 5/6, CK 7, CK 20, TTF1, chromogranin A, synatophysin, NSE, and Ki67. Only samples histologically and immunohistochemically defined as adenocarcinomas of the lung were further evaluated and stained with the rabbit monoclonal anti-LEF1 (Cell Signaling Technology, Boston, USA), anti-TCF4 (Abcam, Cambridge, UK), and mouse anti-β-catenin antibody (Santa Cruz Biotechnology, Heidelberg, Germany). Nuclear LEF1, TCF4, and β-catenin status were evaluated independently by at least two researchers. Nuclear β-catenin and LEF1 expression were classified into four groups: 0 = 0 %, 1+ = 1–25 %, 2+ = 25–50 % and 3+ > 50 % of the tumor cells with positive nuclear staining (Figs. 1, 3). Since nuclear β-catenin and LEF1 expression levels were lower than the TCF4 levels, a different scoring system with five groups was applied for TCF4 staining to discriminate changes between relatively high TCF4 expression levels: 0 = 0 %, 1+ = 1–33 %, 2+ = 33–66 %, 3+ = 66–99 % and 4+ > 99 % (Fig. 2).

### RNA isolation

RNA was isolated with a modified Trizol (Invitrogen, Karlsruhe, Germany) method incorporating a DNase (Roche, Mannheim, Germany) digestion step. Reverse transcription was performed with the iScript Master Mix (BioRad, Munich, Germany) as described previously [14, 15].

### qRT-PCR

Quantitative PCR was carried out as described previously [15] with SYBR Advantage reagents (Clontech, Saint-Germain-en-Laye, France) according to the manufacturer’s instructions using mRNA specific, intron-spanning primers: AXIN2-fwd: GGGCCACTTTAAAGAGCAG, AXIN2-rev:CCTTCATACATCGGGAGCA. All real-time PCRs were performed using the HT 7900 system (Applied Biosystems, Frankfurt, Germany). Ct values for AXIN2, β-catenin, cyclinD1, c-myc, and VEGF-A were calculated using SDS Software Version 2.1 (Applied Biosystems) and gene expressions were normalized to GNB2L1 [15].

### External microarray datasets

Two Affymetrix U133 Plus 2.0 datasets containing 58 primary adenocarcinomas of the lung (GSE3141) and 19 brain metastasis samples of primary adenocarcinomas of the lung (GSE14108) were retrieved from the NCBI Gene Expression Omnibus (GEO) data repository [16]. The primary lung cancer dataset (GSE3141) contained survival information and KRAS mutation status for all samples of early-stage patients; in contrast, the brain metastases dataset did not contain any survival information with respect to the stage IV patients.

### Statistics and bioinformatics

Survival analysis was performed for overall survival since first diagnosis of brain metastasis. Events were defined as cancer-related death; all other events were considered as censored. Survival data were visualized using Kaplan–Meier plots and significance was calculated using the Cox proportional hazards model [17] for univariate and multivariate analyses. Normalized gene expression data of all Affymetrix U133 Plus 2.0 microarrays were log2 transformed. *p* values < 0.05 were considered significant.

To establish LEF1/TCF4 and AXIN2-specific gene signatures, we correlated the expression of selected WNT target genes (Supplementary Table 1) with LEF1 or TCF4 and AXIN2 expression using Pearson`s correlation test in the lung adenocarcinoma brain metastasis dataset (GSE14108). Genes were assigned to the LEF1/TCF4 or AXIN2 gene list according to their mean negative and positive correlations (*r* ≥ 0.4, *r* ≤ −0.4).

The resulting gene signatures were used in hierarchical clustering analyses of the primary lung adenocarcinomas with complete linkage agglomeration, based on the Euclidean distance measure. Differences in overall survival were assessed using Cox proportional hazards regression models. Differential gene expression analysis between patient groups identified by the clustering analysis was performed using empirical Bayes-moderated T-statistics implemented in “limma” [18]. All resulting p-values were adjusted for multiple testing to control the false discovery rate (fdr) using the Benjamini-Hochberg correction.

Gene lists for selected signaling pathways were obtained from the Kyoto Encyclopedia of Genes and Genomes (KEGG) database [19]. Genes were ranked according to the p-value for differential expression from the microarray experiments. In order to test for significantly enriched pathways, a one-sided Wilcoxon rank sum test was performed on the gene ranks for each pathway [20]. All analyses were performed using the free statistical software R (version 2.15.1; http://www.r-project.org).

## Results

### Characterization of the patient cohort

Forty brain metastasis samples of lung cancers were characterized and further classified (see “Materials and methods” section). Twenty-five were defined as adenocarcinomas and included in further investigations. The others proved to be squamous cell carcinoma, small cell lung cancer (SCLC) or unclassifiable and were therefore excluded from this study. Immunohistochemical markers were stained as described in the “Materials and methods” section IHC. The patient cohort was characterized according to the listed parameters in Table 1. Survival was significantly shorter in the cohort with secondary organ metastasis (*p* = 0.02, HR = 3.03). Furthermore the retrospective analysis of radiotherapy (*p* = 0.001, HR = 0.13) as well as chemotherapy after surgery (*p* = 0.005, HR = 0.22) revealed an improved survival (Table 1). The latter results can most likely be attributed to a different performance status of the patients, so that some did not qualify for further post-operative treatment.

**Table 1:** Univariate analysis of clinicopathological baseline data affecting survival

Parameter	Classification	Distribution	Impact on survival hazard ratio [95 % CI]	Impact on survival *p* value (log-rank)
Age	Median [95 % CI]	61 95 % CI [47.8–82.4]	Age ≥61: 1.89 95 % CI [0.78–4.57]	0.1511
Sex	Female (%)	36.0 % (9/25)	Gender male: 2.36 95 % CI [0.82–6.79]	0.1035
	Male (%)	64.0 % (16/25)		
Cerebral metastases present at diagnosis of lung cancer	Yes (%)	72.0 % (18/25)	Present at diagnosis: 1.03 95 % CI [0.37–2.88]	0.9548
	No (%)	28.0 % (7/25)		
Number of cerebral metastases	Solitary (%)	60.0 % (15/25)	Multiple metastases: 0.65 95 % CI [0.26–1.64]	0.3586
	> 1 (%)	40.0 % (10/25)		
Secondary organ metastasis at diagnosis of brain metastasis	Yes (%)	56.0 % (14/25)	Secondary organ metastases: 3.03 95 % CI [1.16–7.95]	0.0178
	No (%)	44.0 % (11/25)		
*CT* Chemotherapy before brain surgery	Yes (%)	16.7 % (4/24^a^)	CT before surgery: 1.50 95 % CI [0.45–5.30]	0.5240
	No (%)	83.3 % (20/24^a^)		
*RT* Radiotherapy of the brain after brain surgery	Yes (%)	87.5 % (21/24^a^)	RT yes: 0.13 95 % CI [0.03–0.54]	0.0010
	No (%)	12.5 % (3/24^a^)		
*CT* Chemotherapy after brain surgery	Yes (%)	66.7 % (12/18^a^)	CT after surgery: 0.22 95 % CI [0.07–0.70]	0.0055
	No (%)	33.3 % (6/18^a^)		
TTF1	Negative ≤3 (%)	32.0 % (8/25)	TTF1 positive: 0.57 95 % CI [0.21–1.52]	0.2565
	Positive >3 (%)	68.0 % (17/25)		
Ki67	Low <10 (%)	32.0 % (8/25)	Ki67 high: 3.27 95 % CI [1.03–10.34]	0.03476
	High ≥10 (%)	68.0 % (17/25)		

Patient cohort was characterized according to listed parameters in the first column. Type of classification and distribution within the cohort as well as impact on survival including *p* value (log-rank) is given for each parameter

^a^Cases where not for all patients baseline data was available

### High proliferation index was associated with shorter survival

**Fig. 4:**
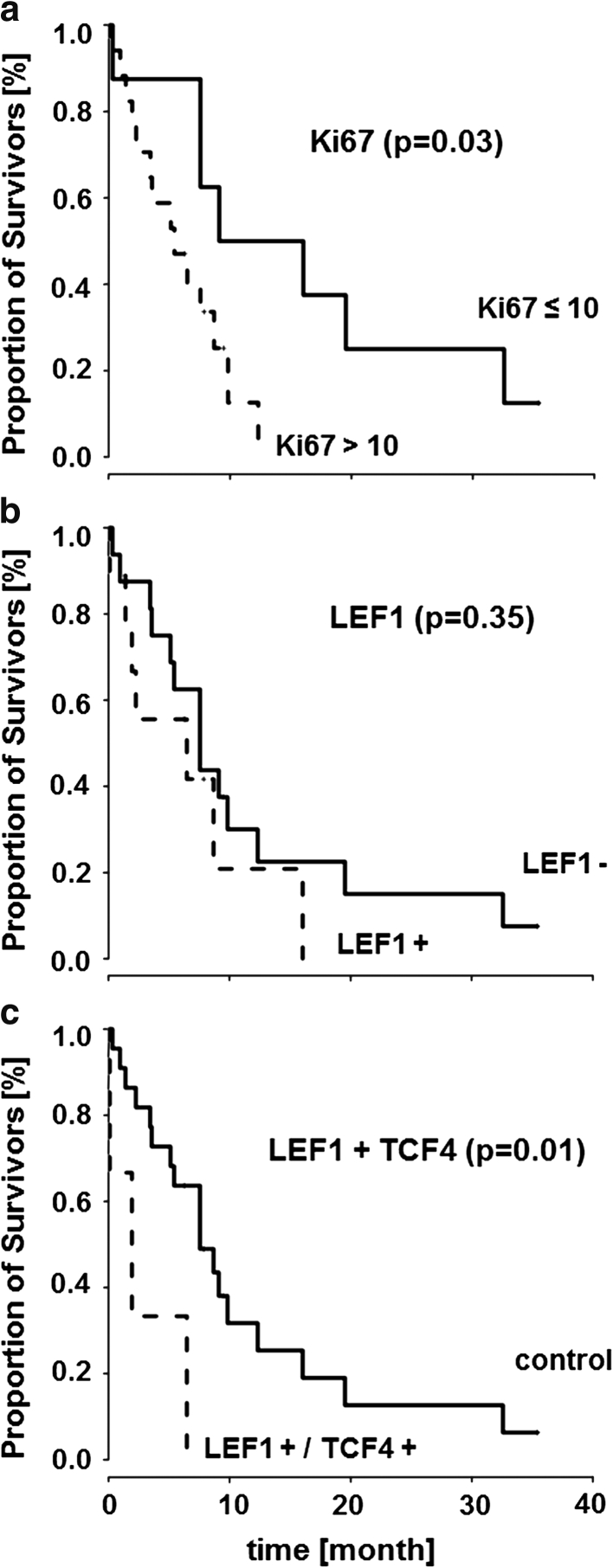
Kaplan-Meier curves illustrate that proliferation index Ki67 (**a**) and LEF1+/TCF4+ (**c**) have a significant impact (Cox proportional hazard ratio) on survival of brain metastasis patients, while no impact can be shown for nuclear LEF1 alone (**b**). Survival is given in months

TTF1 immunostaining was detected in 17/25 (68 %) adenocarcinoma brain metastases. The proliferation index Ki67 ranged from 3 to 40. Seventeen out of 25 samples (68 %) demonstrated a proliferation index of Ki67 > 10 % positive staining. Survival was significantly shorter in the Ki67 > 10 % expressing subgroup of brain metastasis (*p* = 0.03, HR = 3.27) (Table 1 ; Fig. 4a). TTF1 did not have significantly impact survival (*p* = 0.26, HR = 0.57). However, TTF1-negativity correlated with a higher proliferation index Ki67 > 10 % (*p* = 0.02, *r* = −0.47, 95 % CI [−0.73 to −0.09]).

### A subgroup of lung adenocarcinoma brain metastases expressed nuclear LEF1

Nuclear LEF1 immunostaining was detected in 9/25 (36 %) adenocarcinoma brain metastases. Apart from one sample graded 2+, all the others were graded 1+ (see “Materials and methods” section) (Fig. 1). Nuclear TCF4 was expressed in all samples (100 %). Three samples were graded 2+, fifteen samples 3+, and seven samples 4+ (Fig. 2). Nuclear localization of β-catenin was detectable in 9/25 (36 %) samples and ranged from 1+ in seven samples to one sample graded 2+ and 3+, respectively (Fig. 3). Unexpectedly, nuclear localization of LEF1 did not correlate with nuclear β-catenin expression (*p* = 0.53, r = 0.13, 95 % CI [−0.28 to 0.49]). In contrast, nuclear LEF1 correlation with TTF1-negative samples was near the borderline of significance (*p* = 0.06, *r* = −0.38, 95 % CI [−0.66 to 0.02]). A similar tendency with Ki67 > 10 % (*p* = 0.1, *r* = 0.34, 95 % CI [−0.07 to 0.65]) could be observed, while nuclear β-catenin neither correlated with TTF1 (*p* = 0.92, *r* = −0.02, 95 % CI [−0.41 to 0.38]) nor with Ki67 expression (*p* = 0.92, *r* = −0.02, 95 % CI [−0.41 to 0.38]).

### Significantly shorter survival was observed in the LEF1/TCF4 subgroup

Neither TCF4 (*p* = 0.73, HR = 1.19, 95 % CI [0.45 to 3.1]) nor LEF1 (*p* = 0.35, HR = 1.57, 95 % CI [0.61 to 4.03]) (see Fig. 4b) alone demonstrated any significant impact on survival. In contrast, if both nuclear LEF1 and TCF4 (4+) were expressed, survival was significantly shorter (*p* = 0.01, HR = 4.69, 95 % CI [1.23 to 17.9]) (Fig. 4c). Nuclear β-catenin expression had no significant influence on survival (*p* = 0.07, HR = 0.42, 95 % CI [0.16 to 1.09]). Furthermore, no interaction effect with β-catenin could be found for either LEF1 or TCF4.

### AXIN2 expression reflected nuclear β-catenin in lung adenocarcinoma brain metastases

**Fig. 5:**
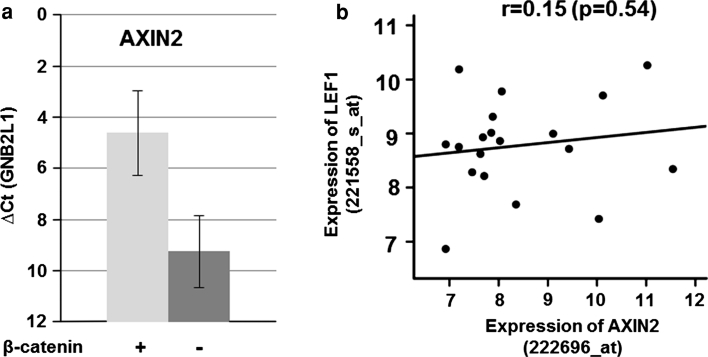
**a** qRT-PCR of AXIN2 in nuclear β-catenin negative and positive samples (*n* = 4). **b** Gene expression of LEF1 and AXIN2 in lung cancer brain metastasis samples of primary adenocarcinomas (GSE14180) revealed no significant correlation (Pearson’s correlation test)

To verify our histological and immunohistochemical findings, namely that expression of nuclear LEF1 and TCF4 are associated with shorter survival and to prove possible β-catenin independence in brain metastasis samples, we analyzed a microarray dataset containing 19 adenocarcinoma brain metastases of the lung. Since gene expression of β-catenin does not reflect nuclear protein localization, we searched for a representative marker, reflecting β-Catenin activity. We therefore quantified the mRNA expression of typical WNT/β-catenin-target genes, such as AXIN2, cyclin D1 and c-myc, in 4 brain metastasis samples by means of qRT-PCR. Among these, AXIN2 correlated best to nuclear β-catenin (Fig. 5a). Gene expression of LEF1 and AXIN2 were correlated in the microarray dataset of the adenocarcinoma brain metastases. However, in line with our histological findings, we found no correlation between LEF1 and AXIN2 gene expression (*p* = 0.54, *r* = 0.15, 95 % CI [−0.33 to 0.56]) (Fig. 5b).

### Coexpression of LEF1/TCF4 and AXIN2 with established WNT-targets differed in cq34567erebral metastasis of lung adenocarcinomas

**Fig. 6:**
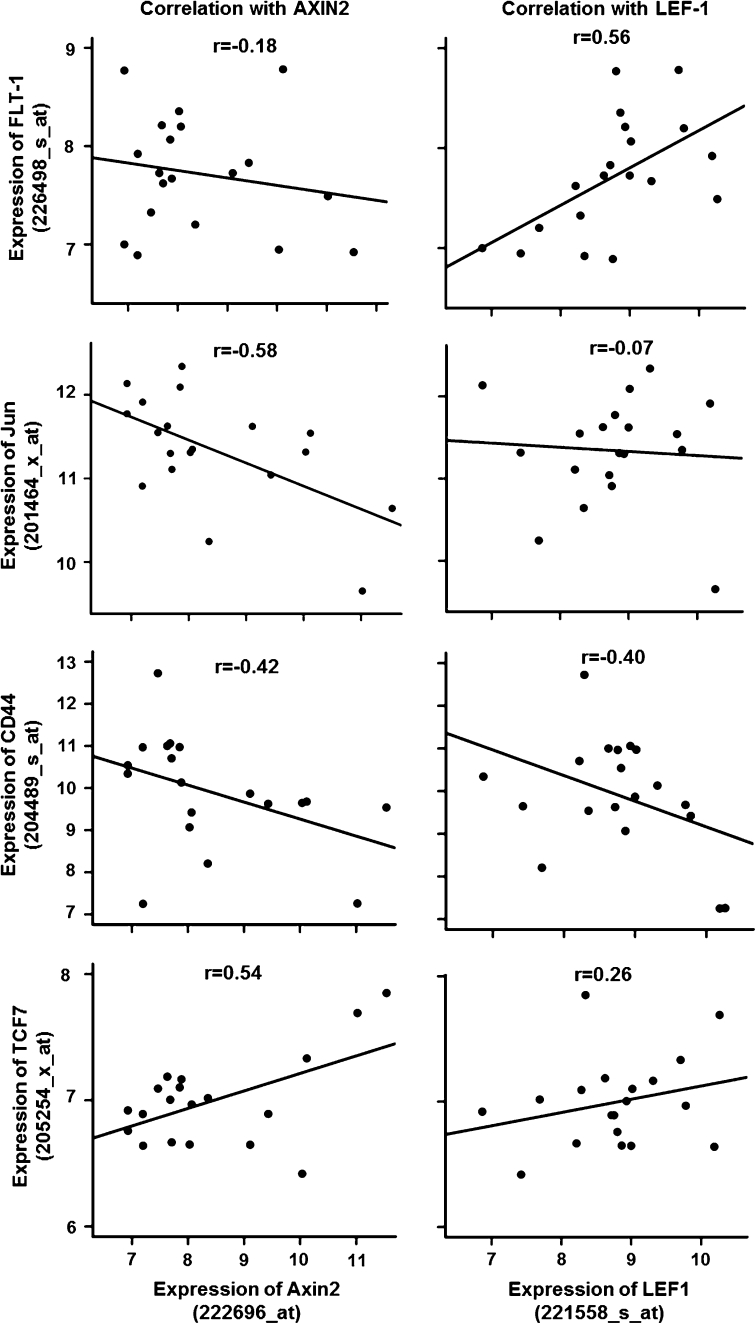
Correlations of LEF1 and AXIN2 with WNT-target genes selected from the WNT-website in lung cancer brain metastases of primary adenocarcinomas of the lung (GSE14180) by Pearson’s correlation test. There is no consistent correlation pattern between LEF1 and AXIN2 with WNT-target gene expression

Subsequently, we correlated the gene expression of LEF1/TCF4 and AXIN2 with 45 established WNT-target genes selected from the WNT website (see Supplemental gene Table 1 with respective probe IDs). There was little overlap between these two cohorts; only a few of the selected target genes were co-expressed concomitantly with both genes (Fig. 6).

Nguyen and colleagues [12] demonstrated an impact of a TCF4 as well as a WNT-lung signature on the relapse of patients with primary lung adenocarcinomas. The authors attributed the effect of LEF1/TCF4 to WNT/β-catenin-signaling. To investigate the interdependency of LEF1/TCF4 and WNT/β-catenin in more detail, we established a LEF1/TCF4 signature as well as an AXIN2 signature, the latter as an indicator for WNT/β-catenin-signaling (for more details see “Materials and methods” section). The LEF1/TCF4 and AXIN2 signature contained *n* = 13/*n* = 12 genes, whereas 45/29 probe IDs of these genes fulfilled the correlation criteria on the microarray, respectively (see Supplemental List 2). Other than CD44 and Gremlin2 (GREM2), the majority of genes were exclusively represented in one of the two signatures.

### LEF1/TCF4 signature defined two clusters with difference in survival in primary lung adenocarcinomas

**Fig. 7:**
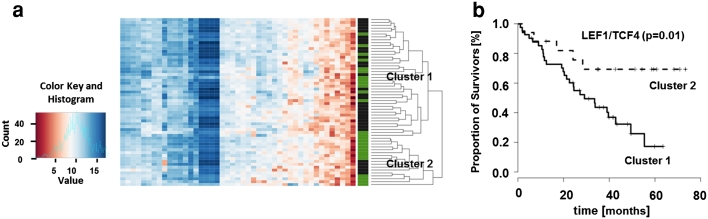
**a** Heatmap of the cluster analysis, based on gene expression values of LEF1/TCF4 signature genes, leads to identification of two separate clusters. *Green bars* mark survivors, *black bars* represent non-survivors. **b** The survival analysis indicates a good prognosis for cluster 2 compared to cluster 1 (Cox proportional hazard ratio of the two clusters). Survival is given in months

To further clarify the predictive power of these two signatures derived from brain metastatic tissue, we tested these in a microarray dataset of 58 early resectable stages of primary lung adenocarcinomas with annotated survival (GSE 14108). Two groups of patients were defined by clustering the gene expression profiles of the LEF1/TCF4 gene signature with significantly different survival (*p* = 0.01, HR = 0.32, 95 % CI [0.12 to 0.83]) (Fig. 7). Further significant differential genes from the microarray data were identified between these two groups.

Only seven genes were significantly differentially regulated, among them CD44 (CD44, HPRT1, LDHA, IGF, 2BP3, B3GALT2, ACACB, ZNF207, and NFYA). Moreover, in the first cluster with poor prognosis all genes except B3GALT2 and ACACB were up-regulated. We then performed comparative gene set enrichment analyses between the two LEF1/TCF4 clusters considering various biological functions (apoptosis, adherens junction, cell cycle, endocytosis, base excision, and nucleotide excision repair) as well as signaling pathways (EGFR-, MAPK-, p53- and WNT signaling) considered to be related to the LEF/TCF family (Table 2). Adherens junction (hsa04520) and cell cycle (hsa04110) were significantly enriched (*p* < 0.01). Within the signaling pathways, WNT and EGFR signaling were not enriched, as opposed to p53 and MAPK signaling. In contrast, the AXIN2 signature was not predictive. Interestingly, none of the previously published β-catenin- [12], TTF1- [21], TCF4- [22] or WNT-lung-signatures [9] was able to either identify major subgroups or to predict survival in this dataset.

**Table 2:** Pathway analyses of the two LEF1/TCF4 clusters in primary lung adenocarcinomas

Pathways	KEGG	*p* value
Adherens junction	Hsa04520	0.002
Apoptosis	Hsa04210	0.068
Base excision repair	Hsa03410	0.060
Cell cycle	Hsa04110	<0.001
Endocytosis	Hsa04144	0.1137
MAPK-signaling	Hsa04010	<0.001
Nucleotide excision repair	Hsa03420	0.026
P53-signaling	Hsa04115	<0.001
WNT-signaling	Hsa04310	0.083

## Discussion

An essential function of the transcription factors LEF1/TCF4 in cerebral metastases of lung adenocarcinomas has been described in mouse models [9], while their clinical relevance in humans is still unclear.

First of all, our data strongly support a role of LEF1/TCF4 in at least one subgroup of cerebrally metastasized adenocarcinoma patients. This subgroup is characterized by a poor prognosis, in particular regarding both nuclear LEF1 and high TCF4 positive (4+) patients. Additionally, we revealed the prognostic relevance of a LEF1/TCF4 signature in primary lung adenocarcinomas in a microarray dataset of primary lung adenocarcinomas. Independently, we demonstrated that Ki67 expression in the metastatic tissues, a recently established marker in early-stage primary lung cancer tissue [8], still has prognostic value in this late stage of the disease. Interestingly, there was a trend in nuclear LEF1 expression to correlate with high proliferation index Ki67. A previous study recently demonstraded that WNT5a expression correlated with a high proliferation index Ki67 and stromal VEGF-A expression, leading to shorter overall survival in primary NSCLC [23]. Interestingly, WNT5a, the typical candidate for β-catenin-independent WNT signaling, can influence the phosphorylation status of various LEF/TCF proteins with effects on gene transcription [24, 25]. Another study demonstrated a correlation of WNT1 with Ki67, c-myc, and poor prognosis in primary NSCLC [26]. All these findings indicate a role of WNT signaling in prognostically unfavorable subgroups of lung cancer. Moreover, all these findings point to cell-type or context-dependent functions of WNT signaling in different subtypes of lung cancer.

Our data are consistent with the different derivation of these tumors from varying cells of origin. The TTF1-negative adenocarcinomas derive from the centrally-based cells: the bronchial basal and the mucous cells [4]. Interestingly, LEF1 (−/−) mice exhibit no submucosal glands derived from central epithelial cells [27], demonstrating a role of LEF1 in centrally-based cell types at least in embryonic development. In contrast, β-catenin (−/−) knock down leads to down-regulation of differentiated peripheral alveolar epithelial cells [28], which are the origin of the peripherally derived TTF1-positive adenocarcinomas. Moreover, constitutively active KRAS and β-catenin in the peripheral Clara cells lead to more aggressive adenocarcinomas by inducing a switch to an embryonic progenitor phenotype, indicating a role of β-catenin in the peripheral cells [29].

Additionally, the cell-type-dependent effects of β-catenin and LEF1/TCF4 are underlined by our cluster analysis results based on the LEF1/TCF4 signature, in which the revealed clusters depicted diverging enrichment patterns of the cell cycle pathway in the primaries of lung adenocarcinomas. Furthermore, none of the previously published β-catenin- [12], TTF1- [21], TCF4- [22] or WNT-lung-signatures [9] was able to either identify major subgroups or to predict survival in this dataset.

This suggests a cell-type-specific function of LEF1 in the central bronchial cells, while β-catenin seems to be more related to the peripheral cells. This may provide an explanation of the lacking correlation between β-catenin and LEF1 in the adenocarcinomas and the differences in the predictive values of the signatures.

However, this does not exclude any direct activation or modification of LEF1/TCF4 by WNT signaling. Further studies are needed to confirm the function of β-catenin, LEF1 and WNT signaling in the two different peripheral (type II pneumocytes, Clara cells) and the two central cell types (basal and mucous) [4].

Cell-type-specific effects of β-catenin and LEF1/TCF4 were already demonstrated in other malignancies. In melanoma, nuclear β-catenin correlates with better prognosis and causes differentiation [30]. In contrast, overexpression of mutated LEF1, lacking the DNA binding domain, results in reduced melanoma cell motility [31]. A study in primary colon cancer described similar discrepancies. In that study, TCF4 overexpression was related to an unfavorable prognosis [32] whereas nuclear β-catenin in late-stage colon cancer predicted a favorable outcome [33].

Taking together the previous results in a mouse model, the defects in embryonic development after LEF1 knock out, and our results, these findings support the importance of LEF1 in a subgroup of lung adenocarcinoma with potential impact on prognosis. However, the mode of its action remains unclear. Despite the lack of correlation with nuclear β-catenin, it could be classical WNT/β-catenin-dependent signaling as suggested in the in vivo study. However, it could also be β-catenin-independent LEF1 or WNT functions [34–36], as suggested for the latter in breast cancer brain metastasis [14, 15]. Either way, β-catenin-independent regulations of the LEF/TCF family members, such as stabilization, localization, and nuclear transport [37–40] as well as their β-catenin-independent architectural function on DNA have only rarely attracted attention in cancer biology to date [10].

In conclusion, this retrospective study underlines the previous results obtained in a mouse model concerning a role of LEF1/TCF4 in brain metastasis of lung adenocarcinoma. Our data indicate that LEF1/TCF4, but not β-catenin, have prognostic relevance in primary and cerebrally metastasized human lung adenocarcinoma patients. However, further studies are needed to highlight the significance of our findings and to specify which signaling pathways are involved in the LEF1/TCF4 function.

## Electronic supplementary material

Below is the link to the electronic supplementary material.

Supplementary material 1 (DOCX 61 kb)

Supplementary material 2 (DOCX 46 kb)
