# Supplementary material for: Nuclear LEF1/TCF4 correlate with poor prognosis but not with nuclear β-catenin in cerebral metastasis of lung adenocarcinomas
Source: Clin Exp Metastasis. 2012 Dec 8;30(4):471–82. doi: 10.1007/s10585-012-9552-7 (PMC3616220; doi:10.1007/s10585-012-9552-7)
Supplement: Supplementary file 1 — Supplementary material 1 (DOCX 61 kb) [file 10585_2012_9552_MOESM1_ESM.docx]

**Supplementary Table 1:** Selected genes from the WNT-website and their corresponding probe IDs on the Affymetrix U133 Plus 2.0 microarray.

| **Affy ID** | **Gene Symbol** |  | **Affy ID** | **Gene Symbol** |  | **Affy ID** | **Gene Symbol** |
| --- | --- | --- | --- | --- | --- | --- | --- |
| 222695_s_at | AXIN2 |  | 212464_s_at | FN1 |  | 211377_x_at | MYCN |
| 222696_at | AXIN2 |  | 214701_s_at | FN1 |  | 234376_at | MYCN |
| 224176_s_at | AXIN2 |  | 214702_at | FN1 |  | 242026_at | MYCN |
| 224498_x_at | AXIN2 |  | 216442_x_at | FN1 |  | 220184_at | NANOG |
| 202094_at | BIRC5 |  | 204420_at | FOSL1 |  | 210673_x_at | NKX2-1 |
| 202095_s_at | BIRC5 |  | 204948_s_at | FST |  | 211024_s_at | NKX2-1 |
| 210334_x_at | BIRC5 |  | 207345_at | FST |  | 231315_at | NKX2-1 |
| 211518_s_at | BMP4 |  | 226847_at | FST |  | 210037_s_at | NOS2 |
| 208711_s_at | CCND1 |  | 203705_s_at | FZD7 |  | 204105_s_at | NRCAM |
| 208712_at | CCND1 |  | 203706_s_at | FZD7 |  | 216959_x_at | NRCAM |
| 1557905_s_at | CD44 |  | 220794_at | GREM2 |  | 210845_s_at | PLAUR |
| 1565868_at | CD44 |  | 235504_at | GREM2 |  | 211924_s_at | PLAUR |
| 204489_s_at | CD44 |  | 240509_s_at | GREM2 |  | 214866_at | PLAUR |
| 204490_s_at | CD44 |  | 201565_s_at | ID2 |  | 208044_s_at | PPARD |
| 209835_x_at | CD44 |  | 201566_x_at | ID2 |  | 210636_at | PPARD |
| 210916_s_at | CD44 |  | 209097_s_at | JAG1 |  | 242218_at | PPARD |
| 212014_x_at | CD44 |  | 209098_s_at | JAG1 |  | 37152_at | PPARD |
| 212063_at | CD44 |  | 209099_x_at | JAG1 |  | 203554_x_at | PTTG1 |
| 216056_at | CD44 |  | 216268_s_at | JAG1 |  | 216994_s_at | RUNX2 |
| 217523_at | CD44 |  | 229924_s_at | JAG1 |  | 221282_x_at | RUNX2 |
| 229221_at | CD44 |  | 231183_s_at | JAG1 |  | 221283_at | RUNX2 |
| 234411_x_at | CD44 |  | 201464_x_at | JUN |  | 232231_at | RUNX2 |
| 234418_x_at | CD44 |  | 201465_s_at | JUN |  | 236858_s_at | RUNX2 |
| 218182_s_at | CLDN1 |  | 201466_s_at | JUN |  | 236859_at | RUNX2 |
| 222549_at | CLDN1 |  | 204584_at | L1CAM |  | 219480_at | SNAI1 |
| 204602_at | DKK1 |  | 204585_s_at | L1CAM |  | 219993_at | SOX17 |
| 1564630_at | EDN1 |  | 221011_s_at | LBH |  | 230943_at | SOX17 |
| 218995_s_at | EDN1 |  | 210948_s_at | LEF1 |  | 203753_at | TCF4 |
| 222802_at | EDN1 |  | 221557_s_at | LEF1 |  | 212382_at | TCF4 |
| 206986_at | FGF18 |  | 221558_s_at | LEF1 |  | 212385_at | TCF4 |
| 206987_x_at | FGF18 |  | 203510_at | MET |  | 212386_at | TCF4 |
| 211029_x_at | FGF18 |  | 211599_x_at | MET |  | 212387_at | TCF4 |
| 211485_s_at | FGF18 |  | 213807_x_at | MET |  | 213891_s_at | TCF4 |
| 214284_s_at | FGF18 |  | 213816_s_at | MET |  | 222146_s_at | TCF4 |
| 231382_at | FGF18 |  | 1566677_at | MMP2 |  | 228837_at | TCF4 |
| 220394_at | FGF20 |  | 1566678_at | MMP2 |  | 205254_x_at | TCF7 |
| 206404_at | FGF9 |  | 201069_at | MMP2 |  | 205255_x_at | TCF7 |
| 239178_at | FGF9 |  | 220541_at | MMP26 |  | 206409_at | TIAM1 |
| 204406_at | FLT1 |  | 204259_at | MMP7 |  | 213135_at | TIAM1 |
| 210287_s_at | FLT1 |  | 203936_s_at | MMP9 |  | 221455_s_at | WNT3 |
| 222033_s_at | FLT1 |  | 202431_s_at | MYC |  | 229103_at | WNT3 |
| 226497_s_at | FLT1 |  | 244089_at | MYC |  | 231743_at | WNT3 |
| 226498_at | FLT1 |  | 203359_s_at | MYCBP |  |  |  |
| 232809_s_at | FLT1 |  | 203360_s_at | MYCBP |  |  |  |
| 1558199_at | FN1 |  | 203361_s_at | MYCBP |  |  |  |
| 210495_x_at | FN1 |  | 209756_s_at | MYCN |  |  |  |
| 211719_x_at | FN1 |  | 209757_s_at | MYCN |  |  |  |
