# Supplementary material for: Nuclear LEF1/TCF4 correlate with poor prognosis but not with nuclear β-catenin in cerebral metastasis of lung adenocarcinomas
Source: Clin Exp Metastasis. 2012 Dec 8;30(4):471–82. doi: 10.1007/s10585-012-9552-7 (PMC3616220; doi:10.1007/s10585-012-9552-7)
Supplement: Supplementary file 2 — Supplementary material 2 (DOCX 46 kb) [file 10585_2012_9552_MOESM2_ESM.docx]

**Supplementary Table 2:** a) LEF1/TCF4 gene signature derived from the brain metastasis dataset with a mean value of (r≥0.4 and r≤-0.4) with all LEF1 or TCF4 probe IDs by correlation. b) The AXIN2 gene signature derived using the same approach.

1. **b)**

| **Gene symbol** | **Affy-ID** |  | **Gene symbol** | **Affy-ID** |
| --- | --- | --- | --- | --- |
| CD44 | 1557905_s_at |  | AXIN2 | 222695_s_at |
| CD44 | 204489_s_at |  | AXIN2 | 222696_at |
| CD44 | 204490_s_at |  | AXIN2 | 224176_s_at |
| CD44 | 209835_x_at |  | AXIN2 | 224498_x_at |
| CD44 | 210916_s_at |  | BMP4 | 211518_s_at |
| CD44 | 212014_x_at |  | CD44 | 1565868_at |
| CD44 | 212063_at |  | CD44 | 204489_s_at |
| CD44 | 217523_at |  | CD44 | 204490_s_at |
| CD44 | 229221_at |  | CD44 | 209835_x_at |
| FGF18 | 214284_s_at |  | CD44 | 210916_s_at |
| FLT1 | 222033_s_at |  | CD44 | 212014_x_at |
| FLT1 | 210287_s_at |  | CD44 | 212063_at |
| FLT1 | 226497_s_at |  | FGF18 | 206986_at |
| FLT1 | 226498_at |  | FGF9 | 206404_at |
| FN1 | 210495_x_at |  | FGF9 | 239178_at |
| FN1 | 211719_x_at |  | GREM2 | 240509_s_at |
| FN1 | 212464_s_at |  | GREM2 | 220794_at |
| FN1 | 216442_x_at |  | GREM2 | 235504_at |
| GREM2 | 235504_at |  | JUN | 201464_x_at |
| GREM2 | 240509_s_at |  | JUN | 201465_s_at |
| GREM2 | 220794_at |  | JUN | 201466_s_at |
| ID2 | 201565_s_at |  | MMP26 | 220541_at |
| ID2 | 201566_x_at |  | PPARD | 210636_at |
| JAG1 | 216268_s_at |  | PPARD | 208044_s_at |
| JAG1 | 209098_s_at |  | PPARD | 37152_at |
| JAG1 | 209099_x_at |  | PTTG1 | 203554_x_at |
| JAG1 | 231183_s_at |  | RUNX2 | 232231_at |
| LEF1 | 210948_s_at |  | TCF7 | 205254_x_at |
| LEF1 | 221557_s_at |  | FGF18 | 231382_at |
| LEF1 | 221558_s_at |  |  |  |
| MET | 203510_at |  |  |  |
| MET | 211599_x_at |  |  |  |
| MET | 213816_s_at |  |  |  |
| MMP2 | 201069_at |  |  |  |
| NRCAM | 204105_s_at |  |  |  |
| SOX17 | 230943_at |  |  |  |
| SOX17 | 219993_at |  |  |  |
| TCF4 | 213891_s_at |  |  |  |
| TCF4 | 203753_at |  |  |  |
| TCF4 | 212382_at |  |  |  |
| TCF4 | 212385_at |  |  |  |
| TCF4 | 212386_at |  |  |  |
| TCF4 | 212387_at |  |  |  |
| TCF4 | 222146_s_at |  |  |  |
| TCF4 | 228837_at |  |  |  |
